# How WHO Solidarity Plus trial participants in countries on four continents are informed in writing

**DOI:** 10.7189/jogh.13.04012

**Published:** 2023-01-20

**Authors:** Rafael Dal-Ré, Teck Chuan Voo, Søren Holm

**Affiliations:** 1 Epidemiology Unit, Health Research Institute-Fundación Jiménez Díaz University Hospital, Universidad Autónoma de Madrid, Madrid, Spain; 2 Centre for Biomedical Ethics, Yong Loo Lin School of Medicine, National University of Singapore, Singapore; 3 Centre for Social Ethics and Policy, Department of Law, School of Social Sciences, University of Manchester, UK

## Abstract

**Background:**

It is unknown if changes have been made to the original participant’s information sheet/informed consent form (PIS/ICF) provided by the WHO Solidarity Plus team when it was transferred to participating countries.

**Methods:**

National principal investigators from 30 countries were asked if the original PIS/ICF was edited in their countries and, if so, to share with us the one used to recruit participants. We assessed whether the 25 different elements of information from the good clinical practice guidelines and the Declaration of Helsinki were present in, deficiently described, or absent from the PIS/ICFs.

**Results:**

Nineteen national principal investigators responded: eight (Argentina, Brazil, Ethiopia, Georgia, Iran, Lebanon, Lithuania, and Malaysia) stated that no edits were introduced to the original PIS/ICF; eight (Canada, Colombia, Philippines, India, Ireland, Pakistan, Portugal, and Switzerland) added some elements of information in the national PIS/ICF; and three (Italy, Peru, and Spain) reported not participating in the trial. None of the elements included in the original PIS/ICF were omitted from the edited PIS/IFC. Six elements of information were omitted and five deficiently described in the original PIS/ICF. The number of elements omitted from the edited PIS/ICFs varied (range = 2-5). Nine PIS/ICFs incompletely described or omitted the informing of study participants about the study results, while five deficiently described or omitted the anticipated expenses for trial participation. Information concerning whom to contact for more information or in case of injury was deficient in six PIS/ICFs. Unlike the original PIS/ICF, all edited PIS/ICFs informed participants about the existence of compensation or treatment for any injury related to the trial.

**Conclusions:**

WHO should consider adding three of the omitted elements in PIS/ICFs of future multinational similar trials.

In 2020 the World Health Organization (WHO) sponsored the Solidarity trial, an open, adaptive trial that assessed four repurposed medications on top of standard of care vs standard of care in hospitalised COVID-19 patients. In August 2021, the WHO announced a second open, large, adaptive trial to assess three new drugs with the same design and in the same patient population, the Solidarity Plus trial. These drugs are artesunate, imatinib and infliximab, already authorized for other indications. Solidarity Plus was to be conducted in 600 hospitals in 52 countries on four continents. Unlike the Solidarity trial, the WHO made the Solidarity Plus trial protocol and other documents (including the participant’s information sheet/informed consent form (PIS/ICF)) freely available from the beginning [1].

There are three basic ethical principles to be fulfilled by any human subject research: respect for persons (or autonomy), beneficence, and justice. Autonomy stands for self-determination and requires protection of those with diminished autonomy. A research study fulfils the principle of beneficence by aiming to produce generalisable knowledge that would be valuable for society and by attempting to maximize benefits and minimize risks for research participants. Finally, justice, in general, requires fairness in participant selection and in the distribution of the study’s benefits and burdens [2]. To fulfil the principle of respect for persons, investigators should seek potential participants’ informed consent prior to their inclusion in any clinical research. The PIS/ICF supports the consent process as it helps with ensuring that all participants have received, at least, the same minimum written information that should be discussed during a conversation with the investigating team member seeking consent. Appropriate language, style, and content are paramount to ensuring its comprehensiveness. The conduct of a multinational trial requires that the original PIS/ICF written by the sponsor be approved by the research ethics committees (RECs) of the participating countries [3,4]. Since RECs must follow national research regulations and should consider the culture and values of the community they serve [5], modifications to the original PIS/ICF text can be expected in one or more of the countries involved in a multinational trial

The PIS/ICFs of the Solidarity and the Solidarity Plus trials are short (four pages) and simple. How these documents are tailored to the context and requirements of clinical research in each participating country is, however, unknown. To our knowledge, the changes introduced to the PIS/ICF by any of the 36 countries participating in the Solidarity trial have not been published. Since the original PIS/ICF of the Solidarity Plus trial was publicly available, we considered that the analysis of the nature and extent of the modifications introduced in the original PIS/ICF in the participating countries would be of interest for future multinational trials sponsored by the WHO or other non-commercial organizations aiming to conduct RCTs in numerous countries across all continents.

## METHODS

To date, the national principal investigators (PIs) of the participating countries in the Solidarity Plus trial have not been disclosed publicly. In June 2022, however, the national PIs of the 36 countries participating in the Solidarity trial became publicly available [6]. We thus decided to include the national PIs of the countries involved in both the Solidarity and the Solidarity Plus trials.

On June 8, 2021, we asked the national PIs of 30 countries whether the original PIS/ICF provided by the Solidarity Plus WHO team was edited when the trial was implemented in their countries, and if so, to share with us the edited version that was being used to recruit participants. The addition of the national/site information was not considered to be an edit of the original text. Three of these 30 PIs asked us to write to the WHO as the global sponsor of the trial. On June 29, the WHO Solidarity Plus team expressed support for our study but made it clear that, although they had the information we were interested in, the information for each country should be provided by the national PIs themselves. On June 29/ 30 and, if necessary, on July 10, we sent reminders to the national PIs of the 30 countries for the requested information noting that the WHO supported our study ─one member of the WHO Solidarity Plus team was copied into all emails sent from June 29 onwards.

The Solidarity Plus trial protocol states that it would be conducted in accordance with the International Conference on Harmonization good clinical practice (GCP) [7]. Compliance with GCP provides “public assurance that the rights, safety and well-being of trial subjects are protected, consistent with the principles that have their origin in the Declaration of Helsinki” (DoH) [8]. Both the DoH [3] and the GCP [8] describe the elements of informed consent that should be included in each trial PIS/ICF which forms the basis of the discussion between potential participants and investigators.

We first reviewed the original PIS/ICF provided by the WHO team and then assessed the PIS/ICF provided by the national PIs who informed us that the document was edited in their countries before starting to recruit participants. Two authors (RDR and TCV) independently reviewed the edited PIS/ICFs included in our analysis to assess whether the 20 elements of information specified in the GCP and the 10 mentioned in the DoH were also mentioned in these PIS/ICFs (**Online Supplementary Document**). These 30 elements were reduced to 25 different elements of information, as five (aims, methods, benefits, risks, voluntariness) are common to both the GCP and the DoH; they were assessed as “yes” (i.e. appropriately described), “no” (i.e. absent), or “deficient” (i.e. incompletely described). Any discrepancies were resolved by discussion between the two reviewers (RDR and TCV).

Finally, we checked the trial protocol and the original PIS/ICF provided by WHO for other relevant information to better understand the informed consent process and the information to be assessed. For instance, the protocol and the original PIS/ICF state that the study would be co-sponsored by the WHO and the National Ministry of Health. These two institutions should provide the study drugs without cost but will not cover any other aspect of patient care [7]. Also, the protocol states that sample size could not be specified at the start of the trial, but that several thousand participants will be recruited and that the WHO would disseminate trial interim and major results [7].

## RESULTS

The national PIs of 16 of the 30 countries responded to our invitation and provided the requested information. Of these, eight (Argentina, Brazil, Ethiopia, Georgia, Iran, Lebanon, Lithuania, and Malaysia) stated that the PIS/ICF used in their countries were translated to the local language, without adding elements of information not already present in the original PIS/ICF provided by the WHO team. The other eight countries (Canada, Colombia, Philippines, India, Ireland, Pakistan, Portugal, and Switzerland), stated the local PIS/ICF were edited and provided us with a copy; all of these were in English except those of Colombia (in Spanish), Portugal (in Portuguese), and Switzerland (in French). The national PIs of Italy, Peru, and Spain informed us that their countries decided not to participate in Solidarity Plus.

Among the eight countries that edited their PIS/ICFs, five (Colombia, Ireland, Pakistan, Philippines, and Portugal) assessed the three medications (artesunate (IV, seven days), infliximab (single IV infusion), imatinib (oral, 14 days)) along with standard of care vs standard of care [7]. Canada is conducting three trials: one assessing these three medications, and two additional trials vs standard of care, one assessing additional administration of dexamethasone and the other assessing LSALT. Finally, two countries (India and Switzerland) assessed only two drugs (infliximab and imatinib). The primary endpoint of the trial is in-hospital mortality [7].

The eight edited PIS/ICFs had between two and 12 more pages than the original PIS/ICF (**Table 1**). Six of the countries that made edits followed the original PIS/ICF with respect to information on sponsorship and financing; however, the Canadian PIS/ICF only mentioned the funder, while the Swiss PIS/ICF mentioned the WHO as both sponsor and funder. Regarding consent options, only the PIS/ICFs from Pakistan and the Philippines followed the original one provided by the WHO team. Notably, in two PIS/ICFs (India and Portugal), there was no possibility of obtaining deferred consent from the participant’s legal representative or relative. Verbal consent option was not permitted in India and Ireland. All eight edited PIS/ICFs identified the involvement of the local REC in the trial approval and stated that a copy of the PIS/ICF would be given to the participant.

**Table 1:** Solidarity Plus trial and information provided by the different versions of the participant’s information sheet and informed consent forms (PIS/ICF) assessed in this study

	PIS/ICF								
	Original WHO, v1.0 April 2021*	Canada, v6.1 May 2022	Colombia, v1.0 April 2021	India, v4.1 October 2021	Ireland, v6.0 April 2021	Pakistan, v1.0 April 2021	Philippines v1.1 August 2021	Portugal, v5.1 June 2021	Switzerland, v6.0 September 2021
**No. of pages**	4	16	10	10	12	6	7	6	6
**Funder (F) or** **sponsor (S)**	WHO (S) and National Ministry of Health (S)	Canadian Institutes of Health (F)	WHO (S) and Ministry of Health and Social Protection (S)	Indian Council of Medical Research (S) and WHO (S)	WHO (S) and Department of Health (S)	Ministry of Health (S) and WHO (S)	WHO (S) and Department of Health (S)	WHO (S) and Agency for Clinical Research & Biomedical Innovation (S)	Swiss National Scientific Fund (F) and the WHO (S & F)
**Consent options**									
From participant’s relative or LAR (deferred consent)	Yes	Yes	Yes	No	Yes	Yes	Yes	No	Yes
Written	Yes	Yes	Yes	Yes	Yes	Yes	Yes	Yes	Yes
Verbal	Yes†	Yes‡	Yes	No	No	Yes	Yes	Yes	Yes
Literate witness§	Yes	No	Yes¶	No	No	Yes	Yes	Yes║	No
Other (phone, mail)	No	No	Yes (email)**	No	Yes (phone)††	No	No	No	No
**Other information provided**									
Review by ethics experts from the WHO‡‡	Yes	No	Yes	Yes	No	Yes	Yes	Yes	No
Local REC mentioned	Yes	Yes	Yes	Yes	Yes	Yes	Yes	Yes	Yes
PIS/ICF copy given to participants or LAR	Yes	Yes	Yes	Yes	Yes	Yes	Yes	Yes	Yes
WHO global liability insurance mentioned§§	No	No	Yes	Yes	Yes	Yes	No	No	Yes

LAR – legally authorized representative, NA – not applicable, REC – research ethics committee, WHO – World Health Organization, PIS/ICF – participant’s information sheet/informed consent form

*Used in Argentina, Brazil, Ethiopia, Georgia, Iran, Lebanon, Lithuania, and Malaysia.

†To be followed by the participant’s consent or LAR consent in a later phase.

‡Verbal consent at the beginning of the trial must be followed by a signed copy of the informed consent form before study completion.

§Because the recruitment of an illiterate participant.

¶Two witnesses are needed.

║Participant must signed the informed consent form as soon as possible.

**By both the participants and the witnesses.

††LAR providing the consent by phone in the presence of a witness.

‡‡The trial protocol was approved on June 23, 2021 by the COVID-19 Research Ethics Review Committee (Geneva; Switzerland) (ISRCTN18066414); §§WHO established a global liability insurance for individuals suffering serious adverse reactions arising from the use of the investigational therapeutics for COVID-19 as part of the Solidarity trial, which will cover all countries and stakeholders that participate in the trial [7].

There are 11 different elements of information that were omitted or deficiently described in the original PIS/ICF provided by the WHO (**Table 2**). None of the original PIS/ICF and the eight edited ones included information on post-study provisions. Mentions of providing the participant with any new available information that could lead him/her to reconsider participation were only present in two edited PIS/ICFs (Canada and Switzerland). Information on when and how study results will be available for participants was deficiently described in all PIS/ICFs and omitted in those of Canada and Switzerland. Funding sources and contacts for trial information and trial injury were incompletely described in seven and six PIS/ICFs, respectively. Anticipated expenses for participants were deficiently described or omitted in five PIS/ICFs. Importantly, all edited PIS/ICFs informed participants about the existence of compensation or treatment for any injury related to the trial, which was not included in the original PIS/ICF.

**Table 2:** Elements of informed consent to be included in the written informed consent form following the guidelines for good clinical practice and the Declaration of Helsinki; only those elements that were omitted or were deficiently included in the original document provided by the WHO team are shown*†

Elements of information	Participant’s information sheet/informed consent form								
PIS/ICF version	Original WHO, v1.0 April 2021‡	Canada, v6.1 May 2022	Colombia, v1.0 Aproi 2021	India, v4.1 October 2021	Ireland, v6.0 April 2021	Pakistan, v1.0 April 2021	Philippines, v1.1 August 2021	Portugal, v5.1 June 2021	Switzerland, v6.0 September 2021
**Good clinical practice (4.8.10)**									
d. Clinical trial procedures	Deficient§	Yes	Deficient§	Yes	Deficient§	Deficient§	Deficient§	Yes	Yes
j. Compensation or treatment available for any injury	No	Yes	Yes	Yes	Yes	Yes	Yes	Yes	Yes
l. Anticipated expenses	No	Yes	Yes	Deficient¶	Yes	No	Deficient¶	Deficient¶	Yes
n. Monitor, auditor, RECs, regulators, have access to participants’ medical records	No	Yes	No	Yes	Yes	No	No	Yes	Yes
p. Providing new information available	No	Yes	No	No	No	No	No	No	Yes
q. Contact person for information and injury	Deficient║	Yes	Deficient**	Yes	Deficient║	Deficient║	Deficient**	Deficient**	Yes
t. Number of participants	No	Deficient††	No	Yes‡‡	No	No	No	Deficient††	Yes ^i^
**Declaration of Helsinki**									
Sources of funding	Deficient§§	Yes	Deficient§§	Deficient§§	Deficient§§	Deficient§§	Deficient§§	Deficient§§	Yes
Possible COI	Deficient¶¶	Yes	Deficient¶¶	Deficient¶¶	No	Deficient¶¶	Deficient¶¶	Deficient¶¶	No
Poststudy provisions	No	No	No	No	No	No	No	No	No
Inform trial results	Deficient║║	No	Deficient║║	Deficient║║	Deficient║║	Deficient║║	Deficient║║	Deficient║║	No

COI – conflicts of interest, REC – research ethics committee, PIS/ICF – participant’s information sheet/informed consent form

*Participant’s information sheet is included as part of the informed consent form.

†All the elements of informed consent included in the guidelines for good clinical practice E6 (R2) and the Declaration of Helsinki are shown in the **Online Supplementary Document**.

‡Original version provided by the WHO team, used in Argentina, Brazil, Ethiopia, Georgia, Iran, Lebanon, Lithuania, and Malaysia.

§Although tests relating to the administration of study drugs are mentioned, information on additional research procedures is lacking.

¶Refers only to study drug costs, but says nothing about other costs.

║Contact for further information is provided but not contact in case of injury/adverse event.

**Contact in case of injury/adverse event is provided but not contact for further information.

††Participants are informed that specific number of participants to be recruited is not known.

‡‡The number of participants to be recruited in the country is provided.

§§Co-sponsors are mentioned, but these could be regarded as the funders by participants as no specific funder was provided.

¶¶Participants are only informed that medical staff will not be paid.

║║Participants are only informed that the findings will be made freely available worldwide.

All elements of information contained in the original PIS/ICF (n = 19) were also incorporated in the eight edited versions included in this analysis. **Table 3** shows that the introduced edits reduced the missing elements of information from 24% in the original PIS/ICF to 8%-20% in the eight edited PIS/ICFs analyzed.

**Table 3:** Assessment of the presence of the 25 different elements of information included in the good clinical practice guidelines and the Declaration of Helsinki

Is the element of information included?	PIS/ICF								
	Original WHO, v1.0 April 2021*	Canada, v6.1 May 2022	Colombia, v1.0 April 2021	India, v4.1 October 2021	Ireland, v6.0 April 2021	Pakistan, v1.0 April 2021	Philippines, v1.1 August 2021	Portugal, v5.1 June 2021	Switzerland, v6.0 September 2021

Yes (present)	14	22	16	19	17	15	15	17	22
Incompletely (deficient)	5	1	5	4	4	5	6	6	0
No (absent)	6	2	4	2	4	5	4	2	3

**Percentage of missing elements**	24	8	16	8	16	20	16	8	12

PIS/ICF – participant’s information sheet/informed consent form

*Used in Argentina, Brazil, Ethiopia, Georgia, Iran, Lebanon, Lithuania, and Malaysia.

**Table 4** shows examples of the appropriate wording used by different PIS/ICFs to describe specific elements of information that were omitted or deficiently described in the assessed PIS/ICFs.

**Table 4:** Wording of specific elements of informed consent that were commonly deficiently described or absent in the participant’s information sheet/informed consent forms (PIS/ICFs) assessed and that could be useful in future multinational trials to be conducted in developing and developed countries

Normative text	Element of information	Text	PIS/ICF of country
GCP	d. The trial procedures to be followed, including all invasive procedures	In general, no additional blood tests, study-specific examinations and additional biological samples are planned for the study.	Switzerland
	j. The compensation and/or treatment available to the subject in the event of trial-related injury.	The sponsor will pay indemnity / insurance for individuals suffering from serious adverse reactions arising from the use of the investigational drugs (…)	Pakistan
	l. The anticipated expenses, if any, to the subject for participating in the trial.	All costs related to the procedures or medicines you receive for your participation will be borne by the study.	Colombia
		There are no costs to participating in this research	Canada
	n. That the monitor(s), the auditor(s), the IRB/IEC, and the regulatory authority(ies) will be granted direct access to the subject's original medical records for verification of clinical trial procedures and/or data, without violating the confidentiality of the subject, to the extent permitted by the applicable laws and regulations (…)	(…) only the representatives of the sponsor, specifically monitors and auditors, and the regulatory authorities will have access to the identifiable data of the participants, preserving the confidentiality and protection of their personal data, in compliance with the applicable legal standard / legislation (…).	Portugal
	p. That the subject or the subject's legally acceptable representative will be informed in a timely manner if information becomes available that may be relevant to the subject's willingness to continue participation in the trial.	Finding during the study: The investigator will notify you, in your capacity as representative, of any new findings that may affect the benefits of the study or the safety and, therefore the consent to participate. You will be informed orally and in writing.	Switzerland
	t. Number of participants	No specific sample size is specified in this public health emergency core protocol. The larger the numbers entered the more accurate the results will be. India may enroll up to (…) COVID cases or more across the country over a period of one year.	India
Declaration of Helsinki	Any possible conflicts of interest	Is there a conflict of interest?: There are no conflicts of interest to declare related to this study. The hospital is being paid a small amount to participate in this study to recover the administration cost of running this study.	Canada

GCP – International Conference on Harmonization good clinical practice, IRB/IEC – institutional review board/independent ethics committee, PIS/ICF – participant's information sheet/informed consent form

** Correspondence to:**  Rafael Dal-Ré, MD, PhD, MPH Unidad de Epidemiología, Instituto de Investigación Sanitaria-Hospital Universitario Fundación Jiménez Díaz, Universidad Autónoma de Madrid, Avda Reyes Católicos 2, 28040 Madrid, Spain rafael.dalre@quironsalud.es

## DISCUSSION

Existing informed consent regulations applicable to countries involved in a multinational trial may not be optimal for helping potential participants make good decisions [9]. This issue could be even more pronounced for trials involving participants who have reduced capacity for decision-making and consequently need to rely on surrogates, as is the case for many Solidarity Plus participants. However, recommendations for addressing specific informed consent issues in critical care research are not often followed [10]. How did the WHO Solidarity Plus team address this when drafting the original PIS/ICF and what did the participating countries do?

To our knowledge, this is the first time that the information provided in PIS/ICFs used to recruit participants in many countries in a WHO-sponsored trial is assessed. We found that in countries from all four continents involved in Solidarity Plus, the original PIS/ICF provided by the WHO was either used without introducing any new element of information or was edited to follow the regulatory or REC requirements of the participating country.

Our assessment of the WHO study team’s original PIS/ICF showed that 24% (6/25) of the elements of information included in the GCP and the DoH were absent; an additional 20% (5/25) were deficiently described. Although eight countries did not edit the original PIS/ICF, the other eight did. Among the latter, the edited PIS/ICFs added several elements of information to reduce the percentage of missing elements to 8%-20% (range = 2-5 of 25). However, not all 25 evaluated elements of information should be considered as relevant to all protocols. Relevant RECs should decide which elements should be included and how they should be described in the PIS/ICFs given the nature of the research and the potential participants’ characteristics.

Although there are several guidance documents on categories of information that should be provided to trial participants [4,11,12], our analysis focused on the 25 different elements of information specified by the GCP [8] and the DoH [3]. The list of 25 elements of information we assessed includes a few more than those contained in the WHO-GCP [13], except for one which informs participants of “the potential risks should confidentiality measures be compromised (e.g. stigma, loss of reputation, potential loss of insurability)”, which does not seem pertinent to Solidarity Plus.

Of most relevance, the original PIS/ICF did not contain information about the WHO establishing a global liability insurance for participants suffering serious adverse reactions from exposure to the investigational medications, covering all participating countries [7]. This was included in the eight edited PIS/ICFs we accessed, indicating the importance of this information. Of utmost importance, five PIS/ICFs did not clearly mention that participation will have no costs for participants. A trial like Solidarity Plus should never entail a cost for participants, regardless of the country where it is conducted. It would be unethical to provide interventional medications free of charge while charging for hospital stay and/or for standard of care drugs or procedures.

The PIS/ICFs of only two countries (Canada and Switzerland) informed the participant that he/she will receive any new information that becomes available that could affect his/her interest to continue in the trial. Since the investigational medications were to be given between one and 14 days, it is unlikely that any new information could impact the course of these treatments. This could be the reason why this information was omitted from most of the PIS/ICFs. However, this cannot be applicable to all participants that are receiving the standard of care, in case an additional medication is incorporated into usual care.

Information on the funding source was deficient in all countries except in Canada and Switzerland. This could be explained by the way the WHO provided this information in the original PIS/ICF, which only mentions co-sponsorship (between WHO and the national Ministry of Health of the participating country), without mentioning any funding source. It is well-known that there is some ambiguity between the meanings of “sponsor” and “funder” [14]. In fact, WHO is reported as funder and co-sponsor in the articles reporting the results of the Solidarity trial [6,15]. Plausibly, using the term “sponsor” interchangeably with “funder” could be acceptable in countries with a poorly developed clinical research culture, though this cannot be the case for countries with well-developed research regulations on informed consent requirements based on international standards.

Strictly speaking, conflicts of interest were deficiently described or absent in all but one PIS/ICF (Canada). Nevertheless, mentioning only that the medical staff would not receive any payment as a form of disclosure of conflict of interest could be considered as sufficient information for participants in countries with limited clinical trial experience, even though receiving income from industry to conduct other studies are of concern in developing countries that usually have low salaries for health care professionals [16]. We understand that the term “conflicts of interest” would likely be difficult to grasp by many participants in developing countries. This is likely, but less so in developed countries, in which other types of conflicts should be considered [17].

Not a single PIS/ICF informed participants on post-study provisions. Since participation in the trial ends at hospital discharge, it could be reasonable to omit this element as it would add no relevant information to potential participants.

Finally, it is difficult to understand why the number of participants to be recruited (in the trial or the country) is not mentioned in the original PIS/ICF or in four of the eight edited documents. Similarly, it is hard to explain that the PIS/ICF in some countries did not mention how the trial results would be provided to participants. We understand that this could be difficult in certain participating countries, but should not be the case in developed countries like Argentina, Canada, Lithuania, and Switzerland.

This study has two main limitations. First, the limited number (n = 16) of participating countries and of those that provided the edited PIS/ICF (n = 8). However, it is difficult to know what percentage it represents of the total number of countries participating in the Solidarity Plus trial. Hence, among the 51 participating countries included on the registry (ISRCTN18066414) as of July 2022, at least three (Italy, Peru, and Spain) are not actually participating. We believe that assessing a larger number of edited PIS/ICF is key to determining the actual status of edits introduced worldwide; however, we believe they are unlikely to be different from what we obtained with the eight countries in our study, which include both countries with developed clinical research system and others lacking it. Second, our assessment may differ from other investigators, particularly on those elements of information that we regard as deficiently described. Yet, this is not relevant, as our main focus is on those elements absent from the assessed PIS/ICFs.

Although the WHO Research Ethics Review Committee issued an informed consent form template for clinical trials [18] (with a length varying depending on the information one would want to provide), the WHO Solidarity Plus team issued a short and simple PIS/ICF. While the use of a tiered approach in developing the PIS/ICF to adapt it to the participants needs has been recommended [19], a reasonable aim is to write the PIS/ICF at a level understandable to the average prospective participant and of a length that can be easily read: people are unlike to read a document of more than 1000 words (four pages) [20]. Since it is very difficult to envisage the average prospective participant in a multinational trial that was to be conducted in settings with large social, economic, cultural, and educational differences, the WHO Solidarity Plus team appears to have taken the approach of including only the minimum (and simple) information that would allow participants to make an informed decision. This approach seems sensible, considering that many potential participants in developing countries will be illiterate and others will have limited understanding of the scientific rationale [16].

## CONCLUSIONS

No elements included in the original PIS/ICF were omitted from the edited ones in the countries, which strongly suggests that, if the WHO team had included one or more of the omitted elements, they very likely would have also been included in the edited ones. We suggest that the original PIS/ICF should have included information on compensation or treatment for injuries that result from exposure to the investigational medicines, the lack of trial-related expenses for participants, and the target number of participants, which would mean adding just a few sentences (**Table 4**). The WHO should facilitate independent assessment of all edited PIS/ICFs of the Solidarity Plus trial, which could inform decisions on which elements of information to include in PIS/ICF provided by WHO teams for future similar trials. This will also be of interest to non-commercial sponsors willing to conduct multinational trials in many continents.

## Additional material


Online Supplementary Document
