# Supplementary material for: How WHO Solidarity Plus trial participants in countries on four continents are informed in writing
Source: J Glob Health. 2023 Jan 20;13:04012. doi: 10.7189/jogh.13.04012 (PMC9850864; doi:10.7189/jogh.13.04012)
Supplement: Online Supplementary Document [file jogh-13-04012-s001.pdf]

Online Supplementary Material

Title: How WHO Solidarity Plus trial participants in countries on four continents are informed

in writing

Authors: Rafael Dal-Ré, Teck Chuan Voo, and Søren Holm

Elements of informed consent

Declaration of Helsinki**.** 64th WMA General Assembly, Fortaleza, Brazil, October 2013.

(https://www.wma.net/policies-post/wma-declaration-of-helsinki-ethical-principles-for-medical-research-involving-human-subjects/)

Informed consent

26. In medical research involving human subjects capable of giving informed consent, each potential subject must be adequately informed of the aims, methods, sources of funding, any possible conflicts of interest, institutional affiliations of the researcher, the anticipated benefits and potential risks of the study and the discomfort it may entail, post-study provisions and any other relevant aspects of the study. The potential subject must be informed of the right to refuse to participate in the study or to withdraw consent to participate at any time without reprisal. Special attention should be given to the specific information needs of individual potential subjects as well as to the methods used to deliver the information.

After ensuring that the potential subject has understood the information, the physician or another appropriately qualified individual must then seek the potential subject's freely-given nformed consent, preferably in writing. If the consent cannot be expressed in writing, the non-written consent must be formally documented and witnessed.

All medical research subjects should be given the option of being informed about the general outcome and results of the study.

Good clinical practice guidelines. (https://ichgcp.net/4-investigator)

4. Investigator

4.8 Informed Consent of Trial Subjects**.**

4.8.10 Both the informed consent discussion and the written informed consent form and any other written information to be provided to subjects should include explanations of the following:

(a) That the trial involves research.

(b) The purpose of the trial.

(c) The trial treatment(s) and the probability for random assignment to each treatment.

(d) The trial procedures to be followed, including all invasive procedures.

(e) The subject's responsibilities.

(f) Those aspects of the trial that are experimental.

(g) The reasonably foreseeable risks or inconveniences to the subject and, when applicable, to an embryo, fetus, or nursing infant.

(h) The reasonably expected benefits. When there is no intended clinical benefit to the subject, the subject should be made aware of this.

(i) The alternative procedure(s) or course(s) of treatment that may be available to the subject, and their important potential benefits and risks.

(j) The compensation and/or treatment available to the subject in the event of trial-related injury.

(k) The anticipated prorated payment, if any, to the subject for participating in the trial.

(l) The anticipated expenses, if any, to the subject for participating in the trial.

(m) That the subject's participation in the trial is voluntary and that the subject may refuse to participate or withdraw from the trial, at any time, without penalty or loss of benefits to which the subject is otherwise entitled.

(n) That the monitor(s), the auditor(s), the IRB/IEC, and the regulatory authority(ies) will be granted direct access to the subject's original medical records for verification of clinical trial procedures and/or data, without violating the confidentiality of the subject, to the extent permitted by the applicable laws and regulations and that, by signing a written informed consent form, the subject or the subject's legally acceptable representative is authorizing such access.

(o) That records identifying the subject will be kept confidential and, to the extent permitted by the applicable laws and/or regulations, will not be made publicly available. If the results of the trial are published, the subject’s identity will remain confidential.

(p) That the subject or the subject's legally acceptable representative will be informed in a timely manner if information becomes available that may be relevant to the subject's willingness to continue participation in the trial.

(q) The person(s) to contact for further information regarding the trial and the rights of trial subjects, and whom to contact in the event of trial-related injury.

(r) The foreseeable circumstances and/or reasons under which the subject's participation in the trial may be terminated.

(s) The expected duration of the subject's participation in the trial.

(t) The approximate number of subjects involved in the trial.
